# Identification and Mechanism of the PD-1/PD-L1 Genomic Signature *SORL1* as Protective Factor in Bladder Cancer

**DOI:** 10.3389/fgene.2021.736158

**Published:** 2021-12-16

**Authors:** Yajing Xu, Didi Chen, Lanxiao Shen, Xiaowei Huang, Yi Chen, Huafang Su

**Affiliations:** ^1^ Department of Radiation Oncology, The First Affiliated Hospital of Wenzhou Medical University, Wenzhou, China; ^2^ Department of Radiotherapy Center, The First Affiliated Hospital of Wenzhou Medical University, Wenzhou, China; ^3^ Department of Oncology-Pathology, Karolinska Institutet, Solna, Sweden; ^4^ Clinical Pathology and Cancer Diagnostics, Karolinska University Hospital, Solna, Sweden

**Keywords:** *SORL1*, bladder cancer, PD-L1, M2 macrophage, EMT, TCGA

## Abstract

**Background:** Immunotherapy has recently shown remarkable efficacy for advanced bladder cancer patients. Accordingly, identifying a biomarker associated with the programmed cell death protein 1 (PD-1)/its ligand (PD-L1) genomic signature to predict patient prognosis is necessary.

**Methods:** In this study, we used mutation data and RNA-seq data of bladder cancer samples acquired from The Cancer Genome Atlas (TCGA) database to combine PD-1/PD-L1-associated mutational signatures with PD-1/PD-L1-associated differentially expressed genes (DEGs). Then, we performed a Kaplan-Meier analysis on the corresponding clinical data of the TCGA bladder urothelial carcinoma (BLCA) cohort to identify prognostic genes, and the results were validated using the GSE48075 cohort. The online platform UCSC Xena was used to analyze the relationship between the candidate genes and clinical parameters. We utilized the Human Protein Atlas (HPA) database to validate the protein expression levels. Then, correlation analysis, cell-type identification by estimating relative subsets of RNA transcripts (CIBERSORT) analysis, and gene set enrichment analysis (GSEA) were used to clarify the mechanism.

**Results:** We identified one prognostic gene, sortilin related receptor 1 (*SORL1*), whose downregulation was associated with a comparatively advanced BLCA stage. While further exploring this finding, we found that *SORL1* expression was negatively correlated with PD-1/PD-L1 expression and M2 macrophage levels. Furthermore, we found that the downregulation of *SORL1* expression was significantly associated with a higher epithelial-mesenchymal transition (EMT) score.

**Conclusion:** We described a novel PD-1/PD-L1-associated signature, *SORL1*, that predicts favorable outcomes in bladder cancer. *SORL1* might reduce immune suppression and inhibit the M2 macrophage-induced EMT phenotype of tumor cells.

## Introduction

Bladder urothelial carcinoma (BLCA) remains the 10th most commonly diagnosed malignancy globally (Sung et al., 2021). Approximately 75% of patients present with nonmuscle-invasive bladder cancer, which has a recurrence rate between 50 and 70%, and the progression rate is approximately 10–30%; the remaining patients have muscle-invasive disease or a metastatic disease and have worse prognoses (Soloway et al., 2002). However, the mortality rate of bladder cancer has been decreasing in recent years due to improvements in treatments, immunotherapy as a representative.

Immunotherapy has gained substantial public attention because it is a promising treatment approach for multiple tumors. In addition, an immune checkpoint inhibitor (ICI) is a powerful weapon for activating therapeutic antitumor immunity (Pardoll, 2012). For example, antibodies against cytotoxic T-lymphocyte antigen 4 (CTLA4), programmed cell death protein 1 (PD-1) and its ligand (PD-L1) target immune checkpoints and activate immune cells to better fight cancers, such as melanoma (Robert et al., 2015; Marzagalli et al., 2019) and non-small-cell lung cancer (NSCLC) (Fehrenbacher et al., 2016; Sun et al., 2020). However, not all tumors are sensitive to immunotherapy, such as pancreatic cancer (Leinwand and Miller, 2020) or glioblastoma (Jackson et al., 2019), and the therapeutic effects of immunotherapy are related to specific characteristics such as tissue specificity (Salmon et al., 2019) and tumor mutational load (Samstein et al., 2019).

For non-muscle-invasive bladder cancer, intravesical immunotherapy with live attenuated bacillus Calmette-Guérin (BCG) is a routine treatment (Lamm, 1992). Evidence has revealed that the resistance of bladder cancer patients to BCG therapy is related to PD-L1 expression (Inman et al., 2007). Although systemic cisplatin-based chemotherapy remains the first-line therapy for advanced bladder cancer, many ICIs have been approved by the Food and Drug Administration (FDA) as a second-line therapy for those with metastatic bladder carcinoma. Some of these drugs have even been used as first-line therapy for cisplatin-ineligible patients due to their remarkable efficacy (Kamat et al., 2016; Lopez-Beltran et al., 2021).

Given the growing importance of immunotherapy for bladder cancer, it is critical to identify an immune-associated biomarker to predict patient prognoses (Zhao et al., 2021; Zhou et al., 2021). Some studies also introduced effective prediction methods to enhanced the understanding of gene expression regulation and therapeutic biomarkers (Zhang L. et al., 2021). The first example we considered was PD-L1 expression levels, as quantified by immunohistochemistry (IHC). However, there are some limitations to measuring PD-L1 expression levels. For example, there is currently a lack of standardization among available PD-L1 assays and cut-offs, and the role of PD-L1 for predicting ICI agent outcomes has yielded mixed results (Xylinas et al., 2014; Bellmunt et al., 2015; Krabbe et al., 2017).

Herein, we integrated PD-1/PD-L1-relevant transcriptome profiles with the mutational signature following the acquisition of accessible RNA-seq data. Then we screened out an immune microenvironment-related gene—*SORL1*—whose expression is related to the prognosis of bladder cancer validated by various methods. We further determined the correlation of *SORL1* with PD-1/PD-L1 expression and M2 macrophage levels, as well as epithelial-mesenchymal transition (EMT) score. Additional exploration on the prognostic signature—*SORL1* which would be a predictive biomarker for bladder cancer need to be performed to see whether it is benefit for patients who were treated with ICI agents.

All in all, we identified a novel genomic signature—*SORL1* that was closely related to immune microenvironment and predicted favorable outcomes in bladder cancer by exploiting public database.

## Materials and Methods

### Data Collection

We obtained publicly available RNA-seq data for bladder cancer patients and the patient clinical information and mutation data from The Cancer Genome Atlas (TCGA) database (https://portal.gdc.cancer.gov). We transformed the RNA-seq data (fragments per kilobase million, FPKM) values into transcripts per kilobase million (TPM) values before performing log normalization. We performed gene annotation using the Ensembl database (http://asia.ensembl.org/biomart/martview). The GSE48075 cohort, which contains 142 bladder cancer samples, was retrieved from the Gene Expression Omnibus (GEO) database (https://www.ncbi.nlm.nih.gov/geo/query/acc.cgi). By removing the samples with a vital state listed as “NA” using the “filter” function in R version 4.0.5 (https://www.r-project.org), we obtained 73 BLCA samples. We obtained a panel of gene signature matrices that distinguished 22 immune cell phenotypes, referred to as LM22, from the available literature (Newman et al., 2015). We also collected a set of 50 hallmark signatures (Liberzon et al., 2015) and a panel of biological function signatures deposited in the Molecular Signatures Database (MSigDB) (https://www.gsea-msigdb.org/gsea/msigdb) to conduct a GSVA analysis.

### Identification of PD-1/PD-L1 Mutational Signatures

We analyzed the PD-1 and PD-L1 mutational signatures in the transcriptome and mutation profiles. For each gene out of a panel containing 16,542 genes, we regrouped 408 patients into mutant and wild-type cohorts. We carried out the Wilcoxon test to observe the differences in PD-1/PD-L1 expression between the mutant and wild-type groups and computed the expression fold change values between these two groups. The genes were only considered PD-1/PD-L1 mutational signatures under the following conditions: 1. mutational frequency >2%; 2. *p* value of Wilcoxon test <0.05; and 3. FC >1.5.

### Identification of PD-1/PD-L1-Associated DEGs

We classified the 408 bladder cancer patients into two groups (high and low expression) using RNA-seq count data. The top 25% PD-1/PD-L1 expression values were treated as a cut-off to differentiate the “high” and “low” expression cohorts. We installed a DESeq2 R Bioconductor package to analyse differentially expressed genes (DEGs) between the two cohorts. The significant DEGs met the criteria of |log2FC|>1.5 and an adjusted *p* value < 0.05. We used the “Enhanced Volcano” R package to draw volcano plots.

### Identification of PD-1/PD-L1 Genomic Signatures

We combined PD-1/PD-L1 mutational signatures with PD-1/PD-L1-associated DEGs to analyze PD-1/PD-L1 genomic signatures using the “draw_venn” function in R.

### Prognosis Analysis for PD-1/PD-L1 Genomic Signatures

Based on the 401 TCGA BLCA samples with available RNA-seq data and clinical information, we performed a Kaplan-Meier analysis of the overall survival (OS) rates to screen genes related to prognosis from the PD-1/PD-L1 genomic signatures. Patients were stratified by gene expression values greater than or below the median score. We applied the log-rank test to observe the differences between the two groups using the “survdiff” function in the “survival” R package, with *p* < 0.05 as the significance threshold. Subsequently, the GSE48075 population was used to test these selected genes for prognostic significance. We adopted the “survminer” R package to plot survival curves.

### Analysis of the Relationship Between *SORL1* Expression and Clinical Parameters

We analyzed the relationship between the *SORL1* expression level and BLCA phenotypes (pathologic T, pathologic N, pathologic M, pathologic stage, primary therapy outcome success, sample type) using the online platform UCSC Xena (http://xena.ucsc.edu/).

### Validation of the Protein Expression of SORL1 in the Human Protein Atlas Database

We determined the protein expression of *SORL1*, both in BLCA and normal tissues, using IHC retrieved from the Human Protein Atlas (HPA, https://www.proteinatlas.org/) (Uhlen et al., 2010).

### Correlation Analysis Between *SORL1* and PD-1/PD-L1 Expression

We analysed the gene expression correlation using the online platform cBioPortal (http://www.cbioportal.org/), which comprises various cancer genomic data. We analysed 408 mRNA expression datasets derived from bladder cancer samples (TCGA, Cell 2017). Additionally, a scatter plot was used to visualize the correlation between the log2-normalized gene expression value of *SORL1* and PD-1/PD-L1, and the *p* value was used to determine statistical significance.

### Determination of Tumor-Infiltrating Immune Cells Associated With *SORL1* Expression

We used the Cell-type Identification by Estimating Relative Subsets of RNA Transcripts (CIBERSORT) method (Newman et al., 2015; Zhang Z. et al., 2021) to quantify the infiltration level of 22 different immune cells. Contrasting the BLCA RNA-seq FPKM matrix and LM22, we estimated the abundances of immune cell types in 401 BLCA patients using the CIBERSORT R script obtained from the CIBERSORT platform (https://cibersort.stanford.edu/) after 1,000 permutations. We excluded the samples with a *p* value for the deconvolution algorithm greater than or equal to 0.05 and retained the remaining 204 samples for further analysis. We conducted a Wilcoxon test to evaluate the differences in immune infiltration levels between the *SORL1* high and low expression groups.

### Identification of Prognosis-Related Significantly Differentially Enriched Signatures

We applied the Gene Set Variation Analysis (GSVA) R Bioconductor package to the reference gene sets to estimate the pathway enrichment scores in different samples (Hänzelmann et al., 2013). We used the limma R package to perform a differential analysis between high and low expression groups divided by the median *SORL1* expression level. We defined the significantly differentially enriched signature based on adjusted *p*-values < 0.001 and |log FC|>0.3.

Then, we performed a Kaplan-Meier analysis and a log-rank test to evaluate the association between the selected differentially enriched signatures and BLCA survival outcomes. *p* < 0.01 was considered statistically significant.

### EMT Scores are Significantly Associated With the Abundance of M2 Macrophages

We obtained an epithelial-mesenchymal transition (EMT) signature enrichment score (defined as the EMT score) from the previous step and computed the M2 macrophage immune infiltration abundance using the CIBERSORT method. We generated the scatter plot of the correlation between the EMT scores and M2 infiltration abundance through the “ggplot2” function in R. We estimated the linear regression line using the “lm” function and estimated the Spearman correlation using the “stat_cor” function in R.

## Results

### Identification of the PD-1/PD-L1 Genomic Signature

**FIGURE 1:**
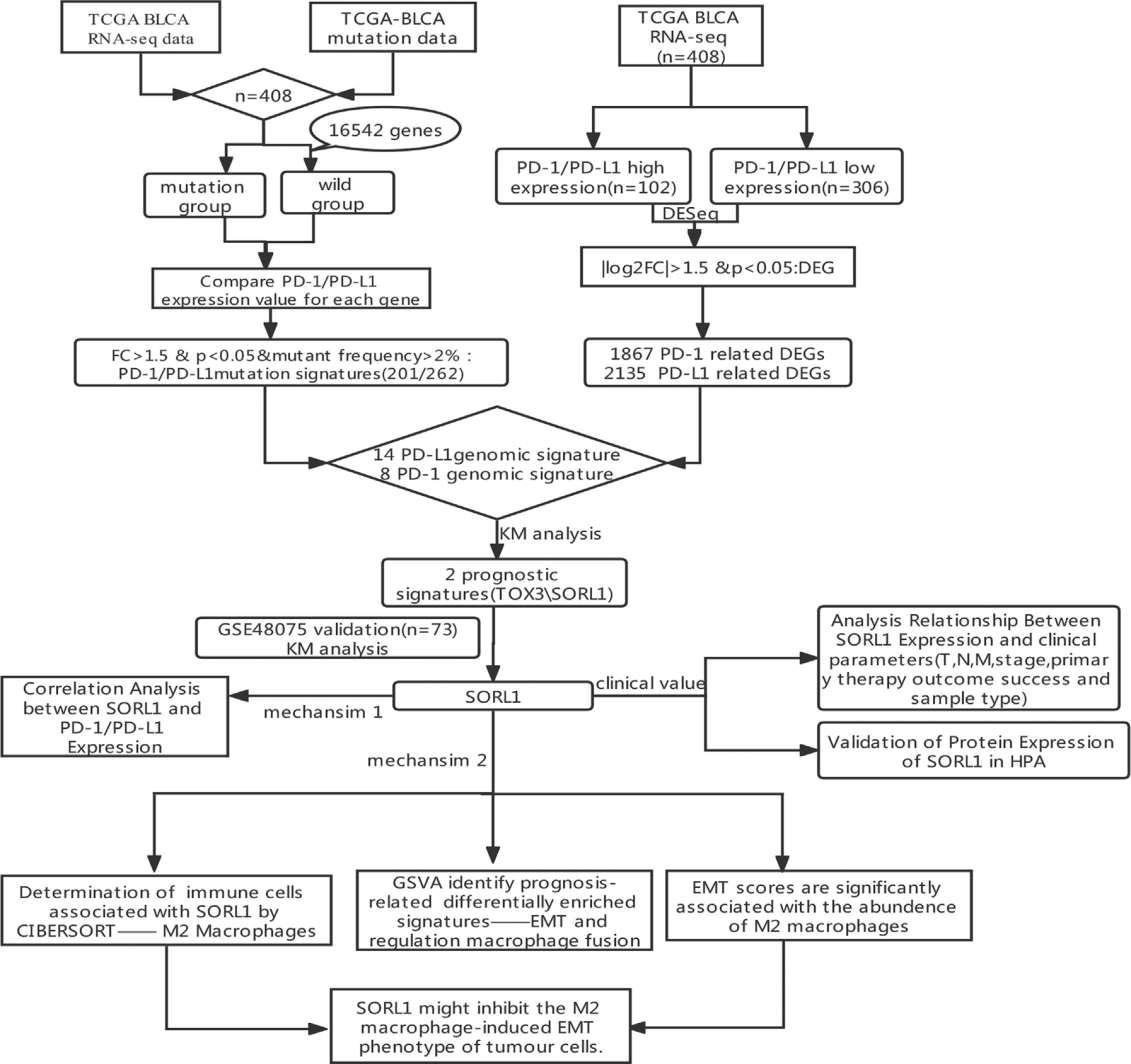
The flowchart of this study.

To identify immune-relevant biomarkers that can predict the prognosis of bladder carcinoma, we explored PD-1/PD-L1-relevant genomic profiles in bladder cancer. Figure 1 shows the flowchart of this study.

**FIGURE 2:**
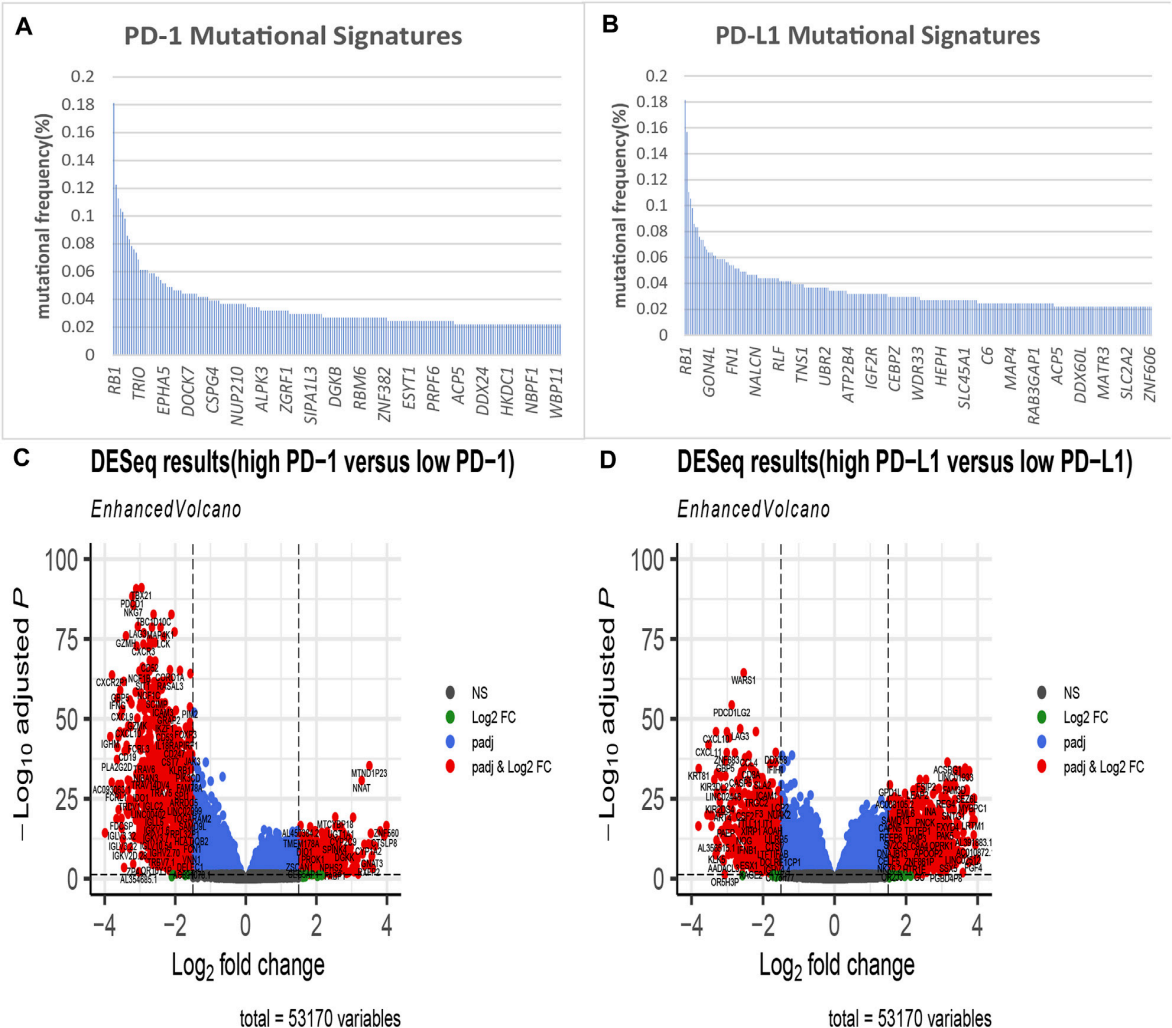
Identification of PD-1/PD-L1 mutational signatures and associated differentially expressed genes (DEGs) in bladder cancer. PD-1 **(A)**/PD-L1 **(B)** mutational signatures of TCGA bladder cancer ranked by mutation frequency (the abscissa labels manifest at random); **(C)** The volcano plot shows 1,867 differentially expressed genes DEGs as red dots between PD-1 high expression samples and PD-1 low expression tissues in bladder cancer (|log2-fold change|> 1.5, *p* < 0.05); **(D)** The volcano plot shows 2,135 differentially expressed genes as red dots between PD-L1 high expression samples and PD-L1 low expression tissues in bladder cancer (|log2-fold change|> 1.5, *p* < 0.05).

We selected 408 bladder tumour patients out of 433 TCGA BLCA samples. These 408 samples were accompanied by both transcriptomic and mutation profile data. We then evaluated the mutation state of 16,542 genes in the selected samples. Their mutation state was studied with a focus on PD-1- and PD-L1-related genomic profiles. For each gene, we regrouped samples into mutant and wild-type cohorts. With the criteria that the mutant group to wild-type ratio had a mean PD-1/PD-L1 expression value >1.5 and a mutational frequency >2%, we obtained the PD-1/PD-L1 mutational signatures listed in Figures 2A,B.

Next, using a minimum |log2FC| of 1.5 and adjusted *p* value of no more than 0.05, we performed a differential expression analysis between the PD-1/PD-L1 high-expression group (102 samples) and low-expression group (306 samples). As a result, we obtained a panel of DEGs out of 53,170 genes, which are described in Figures 2C,D.

**FIGURE 3:**
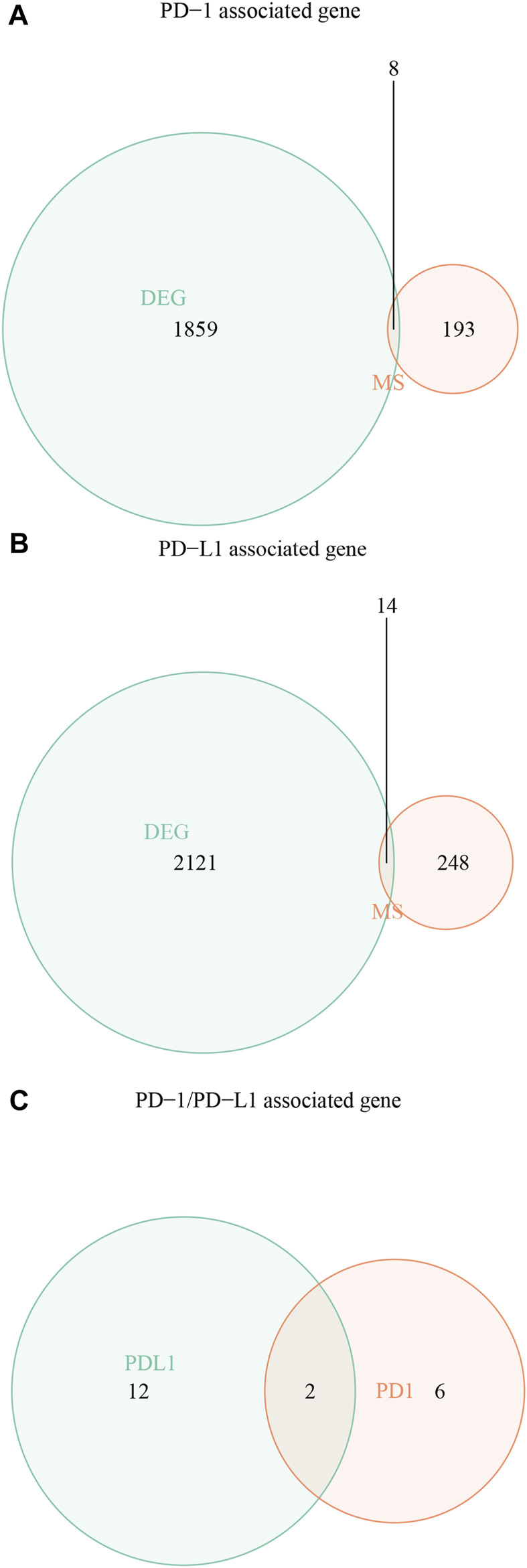
Identification of PD-1/PD-L1 genomic signatures for bladder cancer. **(A)** We obtained a total of 8 PD-1 genomic signatures for bladder cancer by determining the intersection of 201 PD-1 mutational signatures and 1,867 PD-1-associated DEGs. **(B)** In the same way, we acquired 14 PD-L1 genomic signatures determining the intersection of 262 PD-L1 mutational signatures and 2,135 PD-L1-associated DEGs. **(C)** Finally, we obtained 20 overlapping PD-1/PD-L1 genomic signatures.

After Venn analysis, we obtained the PD-1 genomic signatures (*n* = 8) by calculating the intersection of the PD-1 mutational signature and the PD-1-related DEGs (Figure 3A). We carried out the same method for PD-L1 and obtained the PD-L1 genomic signatures (*n* = 14) (Figure 3B). Finally, we combined (*n* = 20) the PD-1- and PD-L1-related signatures (Figure 3C and Table 1).

**TABLE 1:** PD-L1/PD-1 genomic signatures.

Genomic signatures	Belonging to
ITGAL	PD-1
ARHGAP9	PD-1
FERMT3	PD-1
FLT3	PD-1
ZNF560	PD-1
CD163	PD-1
SEZ6L	PD-L1
SORL1	PD-L1
TOX3	PD-L1
HEPHL1	PD-L1
LPA	PD-L1
LRRC31	PD-L1
ERN2	PD-L1
MYO18B	PD-L1
MUC3A	PD-L1
ZNF99	PD-L1
ADAM2	PD-L1
F11	PD-L1
IRS4	PD-1 and PD-L1
SI	PD-1 and PD-L1

### *SORL1* Expression Predicts Favorable Prognosis in Bladder Cancer

**FIGURE 4:**
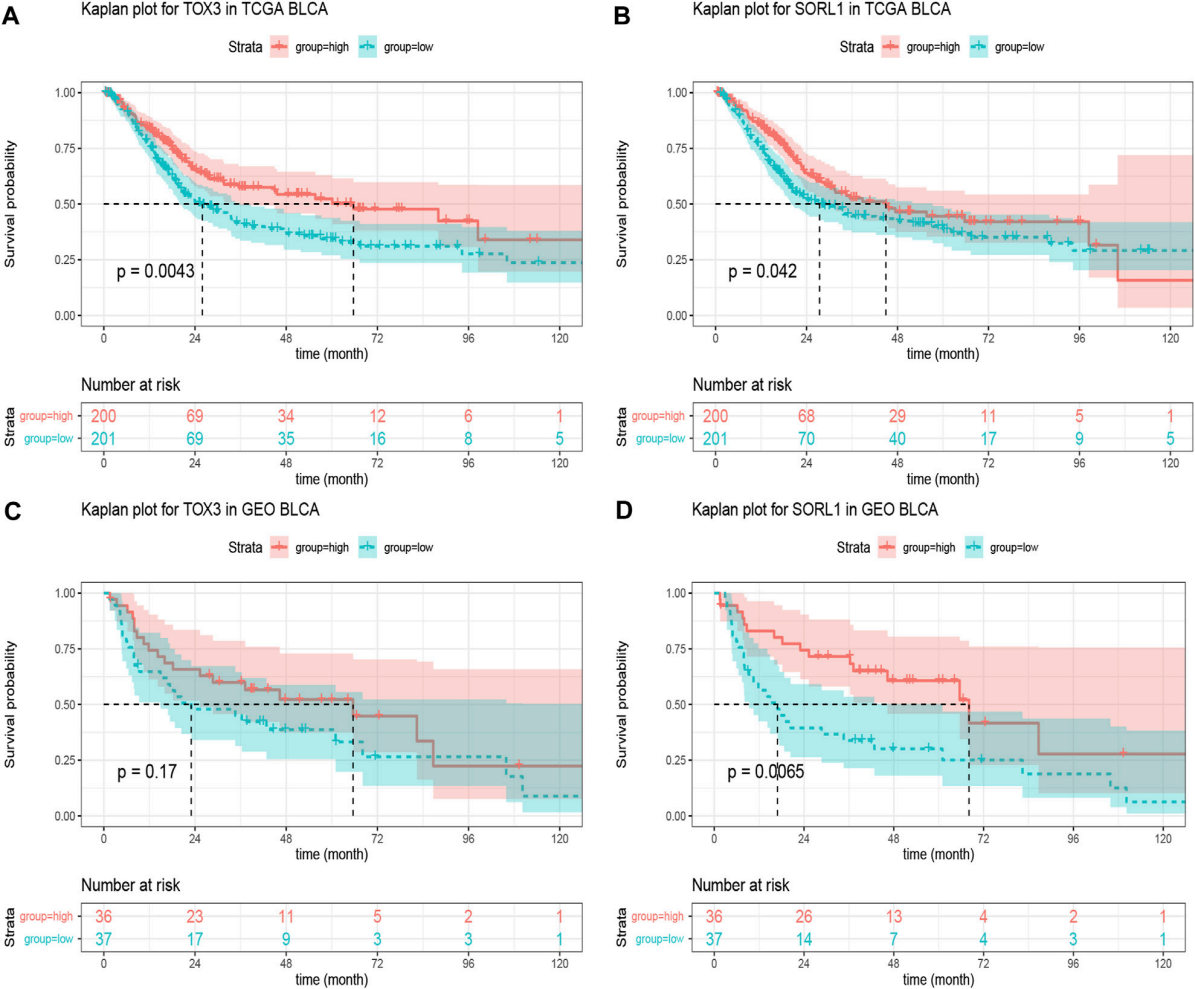
The prognostic assessment of the PD-1/PD-L1 genomic signatures in the TCGA and GSE48075 cohorts. **(A)** Kaplan-Meier curve of overall survival (OS) rates comparing prognosis between *TOX3* high-expression and low-expression groups in the TCGA BLCA cohort; **(B)** OS Kaplan-Meier curve comparing prognosis for *SORL1* high-expression and low-expression groups in the TCGA BLCA cohort. The difference in OS rates between groups with high and low expression levels of *TOX3* **(C)** and *SORL1* **(D)** in the GSE48075 cohort.

Based on the clinical information obtained from the TCGA BLCA samples (401 cases), we performed the Kaplan-Meier survival analysis on 20 genes. Fortunately, we found that the genes *TOX3* (*p* = 0.0043) and *SORL1* (*p* = 0.042) were associated with BLCA (Figures 4A,B). To verify our finding, we applied Kaplan-Meier analysis to the GSE48075 cohort and found that the prognostic value of *TOX3* lacked statistical significance (*p* = 0.17), but that BLCA patients in the *SORL1* high-expression group showed significantly (*p* = 0.0065) more favourable OS outcomes than those in the *SORL1* low-expression group in the GEO validation cohort (Figures 4C,D). In summary, *SORL1* was identified as the only good prognostic indicator for BLCA overall survival outcomes.

### Association of mRNA and Protein Expression of *SORL1* With Clinical Parameters

**FIGURE 5:**
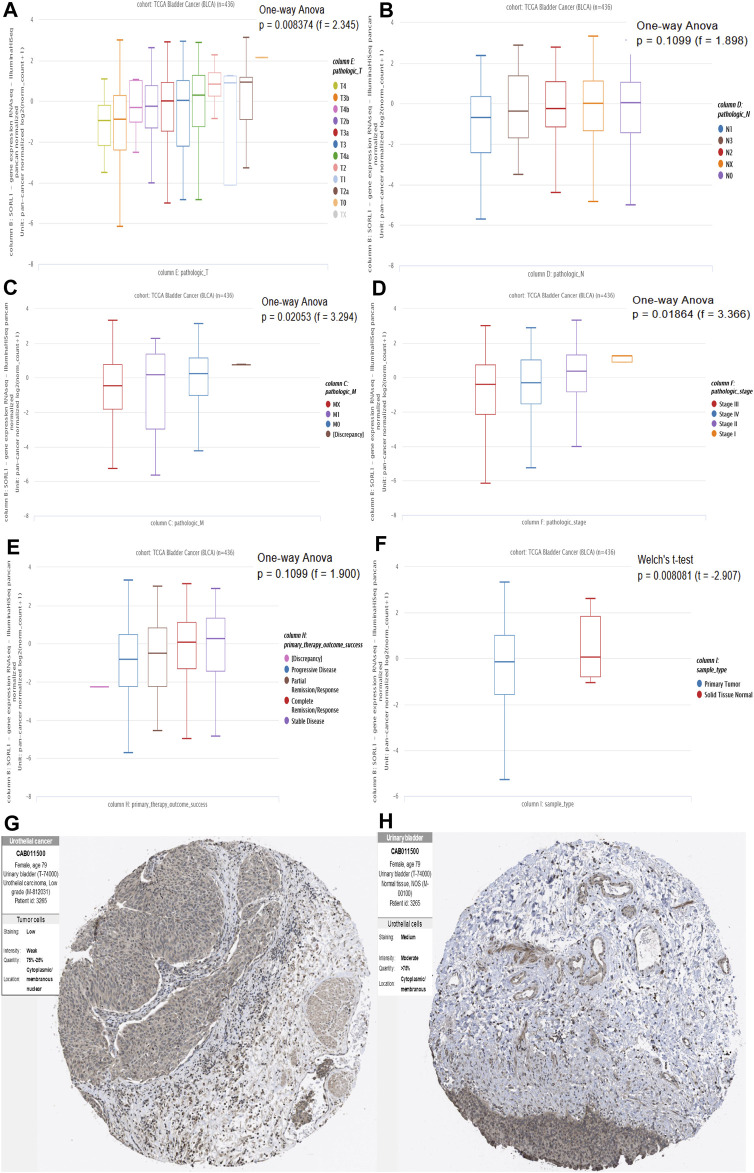
Association of mRNA and protein expression of SORL1 with clinical parameters. Association of SO*RL1* expression levels and pathologic T stage **(A)**, pathologic N stage **(B)** and pathologic M stage **(C)** of BLCA patients; **(D)** The expression of *SORL1* at different pathological stages; **(E)** The influence of the *SORL1* expression level on primary therapy outcome success; **(F)** The expression level of *SORL1* in tumor versus normal tissues in BLCA. **(G)** The protein expression level of SORL1 in BLCA tissues (patient ID: 3265; antibody CAB011500; staining: low; intensity: weak; quantity: 25–75%); **(H)** The protein expression level of SORL1 in normal tissues of BLCA patients (patient ID: 3265; antibody CAB011500; staining: medium; intensity: moderate; quantity: >75%).

After identifying the survival-associated gene *SORL1*, we verified the relationship between the expression levels of *SORL1* and the clinical parameters. From Figure 5A, we found there was a rough trend that the lower the level of *SORL1* expression, the later the T staging (*p* < 0.01). However, there was no statistical significance in N staging (*p* > 0.05) (Figure 5B). Figure 5C shows the association between the *SORL1* expression level and the pathological M stage of bladder carcinoma. A comparatively lower *SORL1* expression level indicated a greater risk for metastasis (*p* < 0.05). Figure 5D depicts a higher expression level of *SORL1* in the earlier stages of bladder cancer (*p* < 0.05). Figure 5E demonstrates the influence of the *SORL1* expression level on primary therapeutic outcomes. However, the influence did not achieve statistical significance (*p* > 0.05). Figure 5F shows that the mRNA expression level of SORL1 was significantly reduced in tumours (*p* < 0.01). Furthermore, the protein levels of the *SORL1* gene were lower in tumour tissues (Figure 5G) than in normal tissues (Figure 5H). Overall, these findings confirmed that the downregulation of *SORL1* expression is associated with worse survival outcomes in BLCA patients.

### Correlation Analysis Between *SORL1* and PD-1/PD-L1 Expression

**FIGURE 6:**
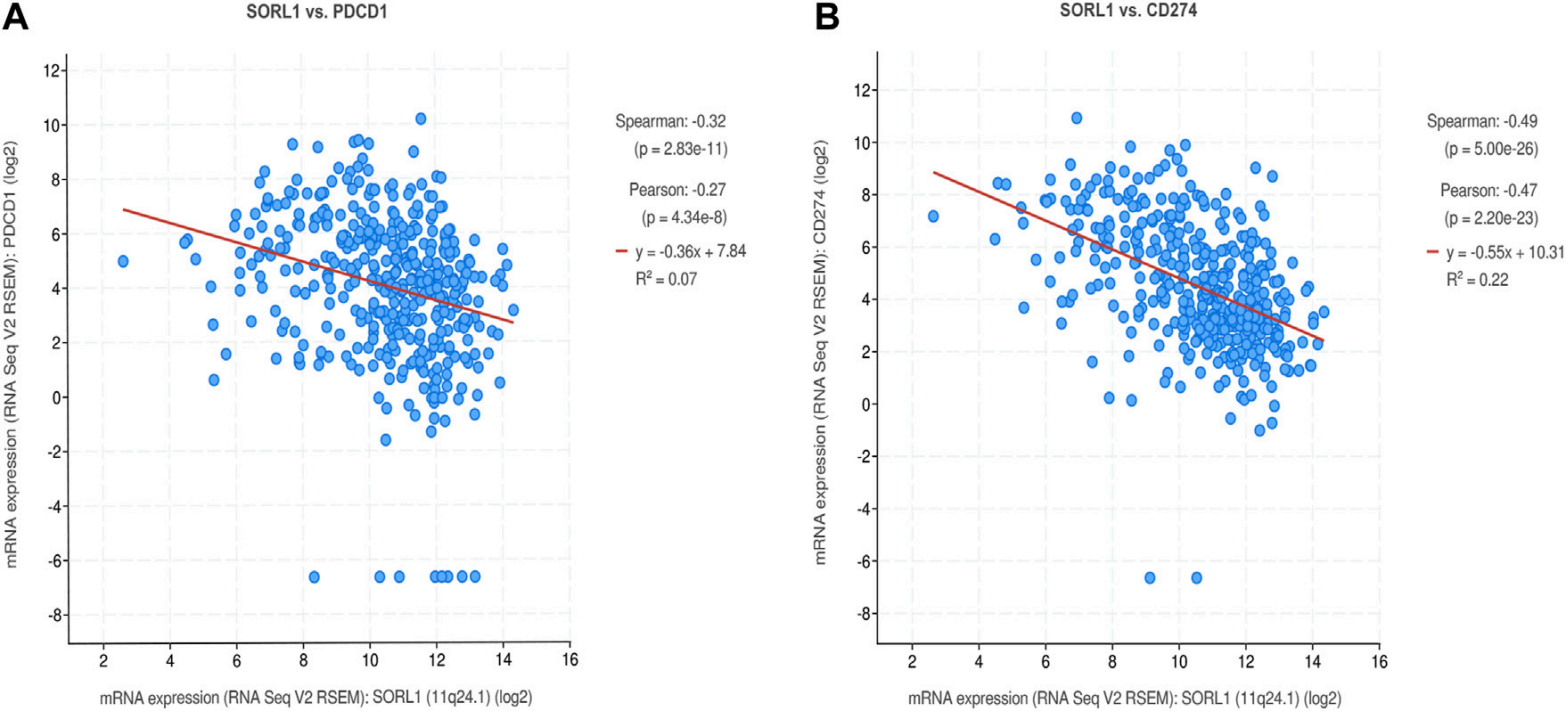
Correlation analysis between SORL1 and PD-1/PD-L1 expression. Correlation scatter plots show the correlation between the expression of PD-1 **(A)**/PD-L1 **(B)** and SORL1.

To investigate the association between the expression of *SORL1* and the immune state, we performed a correlation analysis between *SORL1* and PD-1/PD-L1 expression. We observed a negative correlation between the PD-1 (also named PDCD1) expression value and the PD-L1 (also named CD274) expression value with *SORL1* expression. Additionally, the coefficients of the Spearman correlation were −0.32 (*p* = 2.83e−11) and −0.49 (*p* = 5.00e−26) (Figures 6A,B).

### Determination of Tumor-Infiltrating Immune Cells Associated With *SORL1* Expression

**FIGURE 7:**
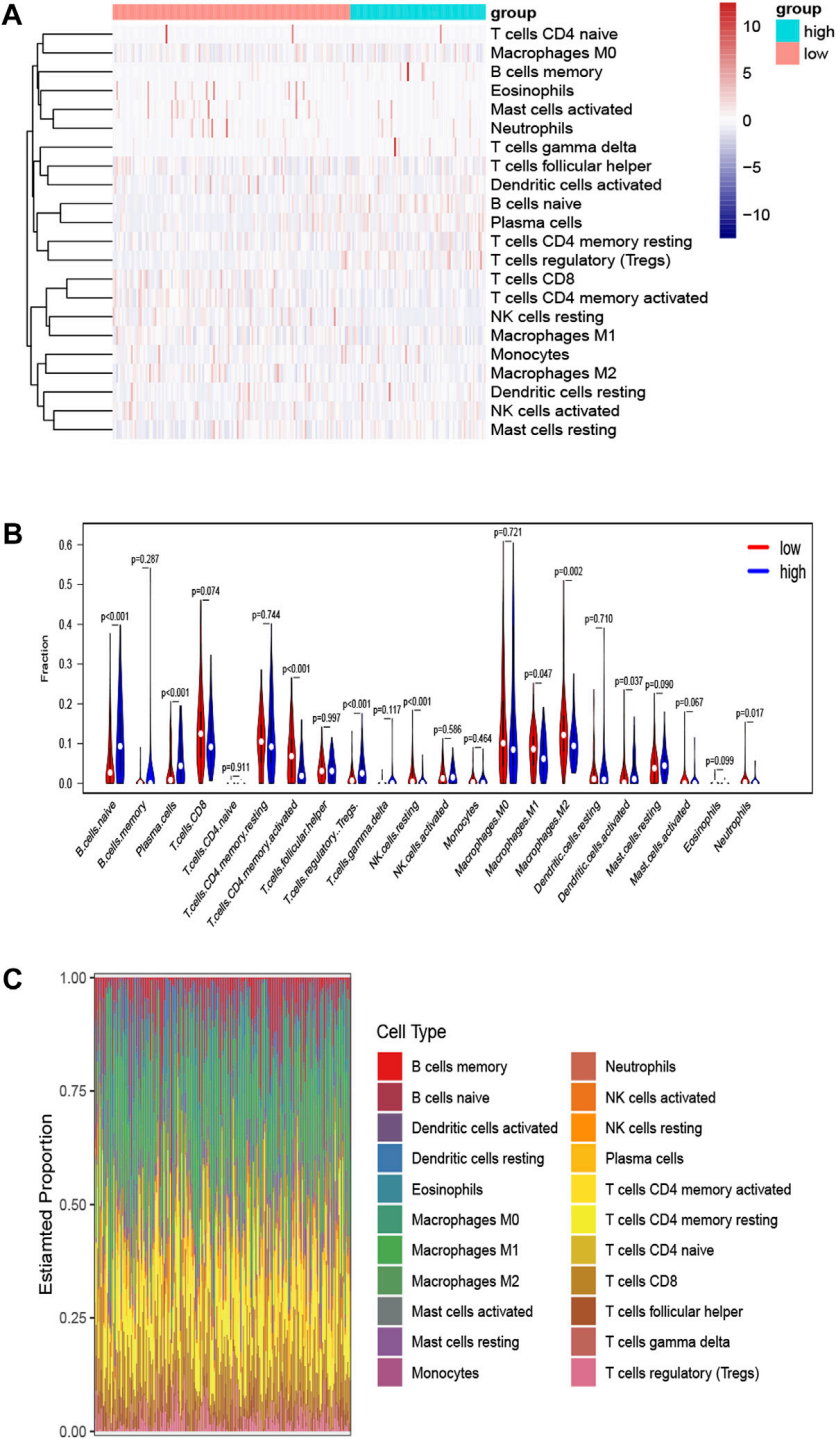
Determination of tumor-infiltrating immune cells associated with SORL1 expression. Heatmap **(A)** and violin plot **(B)** indicate the differences in the 22 immune cell distributions between SORL1 high expression and low expression in the BLCA cohort; **(C)** The histogram shows the landscapes of the proportions of 22 immune cells in the BLCA cohort.

To clarify the survival mechanism related to the relationship between BLCA and the expression of *SORL1*, we explored the differences in tumour-infiltrating immune cells between patients with high and low *SORL1* expression (the group allocation of patients was the same as the previous step). The results are depicted in Figures 7A,B. Figure 7C shows the landscapes of the proportions of 22 immune cells in the selected BLCA patients. We found that the differences in naïve B cells (*p* < 0.001), plasma cells (*p* < 0.001), activated memory CD4 T cells (*p* < 0.001), regulatory T cells (*p* < 0.001), resting NK cells (*p* < 0.001) and M2 macrophages (*p* = 0.002) were statistically significant. Additionally, the differences in naïve B cells, activated memory CD4 T cells, and M2 macrophages were much greater than the others (Figure 7B).

### Identification of Prognosis-Related Significantly Differentially Enriched Signatures

To investigate the *SORL1*-related pathway signatures, we performed GSVA on the TCGA bladder cancer samples.

**FIGURE 8:**
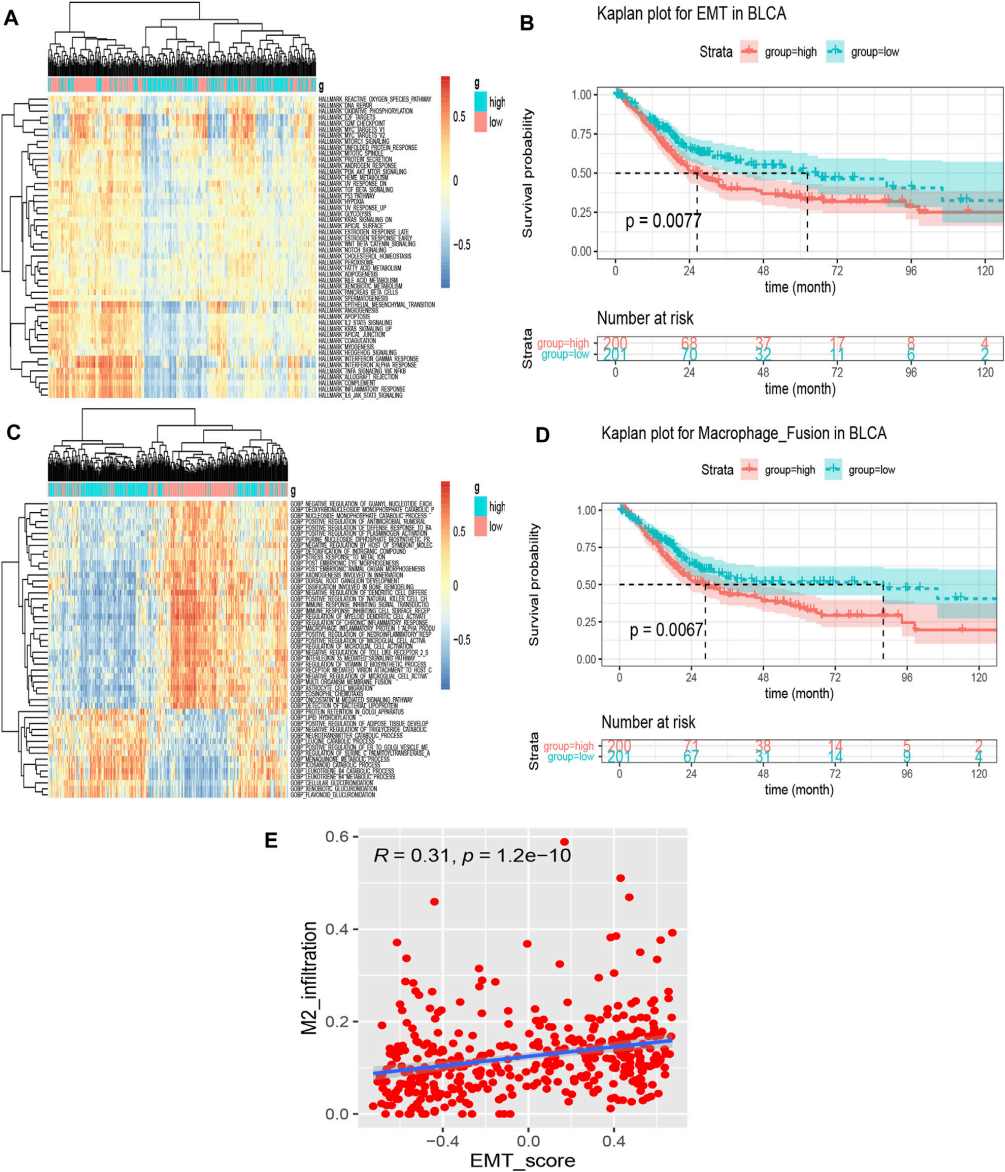
Identification of prognoses related to significant differentially enriched signatures. **(A)** Heatmap of 50 hallmark signature enrichment scores in 401 different bladder cancer samples; **(B)** Kaplan–Meier survival curve for the epithelial-mesenchymal transition (EMT) signature enrichment score based on the TCGA bladder cancer cohort (group cut-off = median); **(C)** Heatmap of the top 50 significantly differentially enriched signatures in the biological process signatures between SORL1 high-expression and low-expression groups by GSVA; **(D)** Kaplan–Meier survival curve for the signature—regulation of macrophage fusion—enrichment score based on the TCGA bladder cancer cohort (group cut-off = median); **(E)** Spearman’s correlation between the EMT score and M2 macrophage level.

Using GSVA, we obtained the enrichment scores of 50 hallmark signatures approximately in a normal distribution. We presented the association between the hallmark signatures and *SORL1* expression level in the form of a heat map (Figure 8A). We identified three significantly differentially enriched signatures between the *SORL1* high-expression and low-expression groups using the criteria of adjusted *p*-values < 0.001 and |log FC|>0.3. These were HALLMARK_ALLOGRAFT_REJECTION, HALLMARK_EPITHELIAL_MESENCHYMAL_TRANSITION and HALLMARK_INFLAMMATORY_RESPONSE. Subsequently, the Kaplan-Meier analysis showed that the pathway signature EMT was associated with significantly worse survival outcomes (*p* = 0.0077) (Figure 8B).

For a set of biological process signatures, we also obtained 251 significantly differentially enriched signatures using the same criterion as the one described above. Figure 8C shows the top 50 differentially enriched pathways. We also applied Kaplan-Meier analysis to these 251 signatures and found that 14 signatures were associated with BLCA patient prognosis (Table 2). When we focused on the results, the signature regulation of macrophage fusion caught our attention. This signature was associated with poor prognosis among bladder cancer patients (*p* = 0.0067), as shown in Figure 8D.

**TABLE 2:** 14 prognostic pathway signatures of biological processes associated with *SORL1* expression in the TCGA bladder cancer cohort.

Number	Biological processes signatures
1	GOBP_REGULATION_OF_ANTIBACTERIAL_PEPTIDE_PRODUCTION
2	GOBP_POSITIVE_REGULATION_OF_PLASMINOGEN_ACTIVATION
3	GOBP_PEPTIDYL_LYSINE_HYDROXYLATION
4	GOBP_CHONDROITIN_SULFATE_BIOSYNTHETIC_PROCESS
5	GOBP_REGULATION_OF_MACROPHAGE_FUSION
6	GOBP_OSSIFICATION_INVOLVED_IN_BONE_REMODELING
7	GOBP_NEGATIVE_REGULATION_OF_PROTEIN_KINASE_ACTIVITY_BY_REGULATION_OF_PROTEIN_PHOSPHORYLATION
8	GOBP_NEGATIVE_REGULATION_BY_HOST_OF_SYMBIONT_MOLECULAR_FUNCTION
9	GOBP_MODULATION_BY_HOST_OF_SYMBIONT_MOLECULAR_FUNCTION
10	GOBP_CELLULAR_RESPONSE_TO_CADMIUM_ION
11	GOBP_CELLULAR_RESPONSE_TO_HEPARIN
12	GOBP_POSITIVE_REGULATION_OF_ADIPOSE_TISSUE_DEVELOPMENT
13	GOBP_POSITIVE_REGULATION_OF_GONAD_DEVELOPMENT
14	GOBP_DORSAL_ROOT_GANGLION_DEVELOPMENT

### EMT Scores are Significantly Associated With the Abundance of M2 Macrophages

To further explore the correlation between EMT and M2 macrophages in bladder carcinoma, we performed a correlation analysis on the EMT score and M2 infiltration abundance. Figure 8E depicts a significant positive correlation between M2 macrophage levels and the EMT signature (cor = 0.31, *p* = 1.2e−10).

## Discussion

Scientists widely acknowledge that immunotherapy is remarkably efficient for advanced bladder cancer, the most common malignancy of the urinary system (Kamat et al., 2016; Lopez-Beltran et al., 2021). Therefore, it is necessary to identify an immune-related biomarker to predict the prognosis of BLCA patients. In this study, we explored the genomic alterations present in high-expression PD-L1 and PD-1 samples, as well as the differentially expressed genes between PD-L1/PD-1 high- and low-expression samples. Herein, we aimed to seek out immune-related genomic signature predicting survival outcome of bladder cancer and attempted to explore its value as biomarker in clinical practice.

Firstly, we selected the PD-1/PD-L1 mutational signatures from 16,542 genes when their mutational frequency and the fold change value of mutant to wild met our expectation. Later we performed differential expression analysis between the top 25% of PD-1/PD-L1 expression and the remaining low expression samples, further screening condition of |log2FC| ＞1.5 with statistical significance determined PD-1/PD-L1 -associated DEGs. By analyzing the overlapping genes, we fortunately identified two immune microenvironment-related gene—sortilin related receptor 1 (*SORL1*) and TOX high mobility group box family member 3 (*TOX3*).

It is worth noting that a minor allele of *TOX3* has been widely reported to implicate in an elevated risk of breast cancer (Stevens et al., 2011; Man et al., 2020) and be associated with the prognosis of patients (Fasching et al., 2012). Besides, research found *TOX3* may play a vital part in the prognosis and treatment of gastric cancer (Zhang et al., 2013) as well as be a therapeutic target for clear renal cell carcinoma (Jiang et al., 2019) and colorectal cancer (Saleh et al., 2020). In TCGA database, it also predicts favorable survival outcomes for BLCA samples with *p* < 0.05, nevertheless, this underlying biomarker was denied by GSE48075 validation cohort (*p* > 0.05). Therefore, *SORL1* is the only remaining genomic signature with prognostic value.

*SORL1*, also known as *SORLA* or *LR11*, encodes a protein belonging to the vacuolar protein sorting 10 (VPS10) domain-containing receptor family and the low-density lipoprotein receptor (LDLR) family. We found that mutation of this gene is associated with Alzheimer’s disease (Campion et al., 2019) and hematologic neoplasms (Sakai et al., 2012; Kawaguchi et al., 2013; Ohwada et al., 2015). According to Pietilä et al. (2019), the depletion of *SORL1* triggers HER2 accumulation in dysfunctional lysosomes, and *SORL1*-silenced cells showed antiproliferative effects on not only breast cancer but also bladder cancer by regulating oncogenic receptor tyrosine kinase (RTK) signaling. This group further investigated the role of *SORL1* in mediating targeted therapy resistance in breast cancer and discovered that *SORL1* is necessary for HER2-HER3-driven oncogenic cell growth (Al-Akhrass et al., 2021). However, Michael et al. (2019) discovered that the downregulation of *SORL1* expression facilitates tumor growth in a transplant tumor model and relatively low expression predicts a worse prognosis in human breast, lung, and gastric cancer patients. However, we have yet to determine the precise mechanism of this finding.

The results of our study indicate that *SORL1* is a protective factor in bladder cancer. A comparatively lower *SORL1* expression level increased metastasis risk and was associated with relatively advanced disease stages, while a higher expression level of *SORL1* was associated with a favorable prognosis in BLCA patients. The difference in protein expression levels between tumor and normal tissues further demonstrates the reliability of our analysis.

We selected *SORL1* from the PD-1/PD-L1-associated genomic signature. Consistently, a negative correlation was observed between the expression level of *SORL1* and that of PD-1/PD-L1. PD-1 and PD-L1 are immunosuppressive molecules. Therefore, the upregulation of *SORL1* expression inhibited the expression of PD-1/PD-L1 and then reduced the state of immune suppression, reactivating immune cells and allowing them to attack tumor cells. This may explain why *SORL1* is a protective factor against bladder cancer.

Another reason for the protective role of *SORL1* is that BLCA patients with high *SORL1* expression generally showed lower M2 macrophage infiltration levels. GSVA also indicated that regulation of macrophage fusion was a significantly differentially enriched signature associated with *SORL1* expression levels. Previous research indicated that M2 macrophages promote angiogenesis, tumor progression and metastasis (Pollard, 2004). Although few studies have covered M2 macrophage’s potential protective effects on bladder cancer (Aljabery et al., 2018), M2 macrophages have been associated with poor survival outcomes for bladder cancer patients in most studies (Ayari et al., 2009; Maniecki et al., 2012; Suriano et al., 2013; Martínez et al., 2017), along with in ours, as shown in Figure 8D. Additionally, GSVA revealed that comparatively lower *SORL1* expression is associated with a higher EMT enrichment score, a cellular process during which epithelial cells acquire mesenchymal characteristics as a result of suppression of epithelial phenotypes (Yang et al., 2020). Studies have revealed that macrophages participate in EMT induction (Hu et al., 2021; Yang et al., 2021) and that EMT plays an important role in tumor metastasis and progression, including in bladder cancer (McConkey et al., 2009). Our study also indicated a positive correlation between M2 macrophages and the EMT score. Although no studies have revealed a direct relationship between EMT and M2 macrophages and the prognosis of bladder patients to date, we presume that the upregulation of *SORL1* expression may predict a better prognosis among bladder patients as a result of *SORL1*-mediated inhibition of the M2 macrophage-induced EMT phenotype of tumor cells.

Although we identified a PD-1/PD-L1 genomic signature, *SORL1*, with favorable prognostic value for bladder cancer patients and explored the possible mechanism, there are several limitations in this study. First, almost all BLCA cohorts were mined from the TCGA database. Thus, we should expand our sample size to include different databases to verify our findings. Second, the comprehensive mechanism by which *SORL1* affects BLCA prognosis remains unclear, besides, the detail of gene regulatory network for *SORL1* which some literatures have introduced remains unexplored (Liu et al., 2021). Thus, further experimental exploration may contribute to providing direct evidence. Finally, the effects of the *SORL1* gene on immunotherapy among bladder cancer patients in the real world are unclear.

## Conclusion

In summary, we herein described a novel PD1/PD-L1-associated signature, *SORL1*, that was associated with favourable outcomes among bladder tumour patients. *SORL1*, as a protective factor, may alleviate the state of immune suppression and inhibit the M2 macrophage-induced EMT phenotype of tumour cells.

## Data Availability

The original contributions presented in the study are included in the article/Supplementary Material, further inquiries can be directed to the corresponding authors.

## Abbreviations

BCG, bacillus Calmette-Guérin; BLCA, bladder urothelial carcinoma; CIBERSORT, cell-type identification by estimating relative subsets of RNA transcripts; CTLA4, cytotoxic T-lymphocyte antigen 4; DEGs, differentially expressed genes; EMT, epithelial-mesenchymal transition; FDA, Food and Drug Administration; FPKM, fragments per kilobase million; GEO, Gene Expression Omnibus; GSVA, gene set variation analysis; HPA, Human Protein Atlas; ICI, immune checkpoint inhibitor; IHC, immunohistochemistry; LDLR, low-density lipoprotein receptor; MSigDB, Molecular Signatures Database; NSCLC, non-small-cell lung cancer; OS, overall survival; PD-1/PD-L1, programmed cell death protein 1/its ligand; RTK: receptor tyrosine kinases; SORL1, sortilin related receptor 1; TCGA, The Cancer Genome Atlas; TPM, transcripts per kilobase million; VPS10, vacuolar protein sorting 10.
